# Night eating behavior, sleep quality, body composition, and type 2 diabetes risk among Saudi Arabian females: a cross-sectional study

**DOI:** 10.1038/s41598-026-40702-4

**Published:** 2026-02-23

**Authors:** Wafa Alotaibi

**Affiliations:** Department of Food and Nutrition Science, College of Agricultural and Food Science, King Faisal University, 31982 Al-Ahsa, Saudi Arabia

**Keywords:** Night eating, Sleep quality, Body composition, Type 2 diabetes risk, Chrononutrition, Diseases, Endocrinology, Health care, Medical research, Risk factors

## Abstract

Night eating behavior has been linked to circadian disruption and adverse metabolic outcomes, yet evidence remains inconsistent, particularly in young and metabolically healthy populations. This cross-sectional study examined associations between night eating behavior, sleep quality, body composition, and type 2 diabetes risk among 150 Saudi Arabian females. Night eating severity, sleep quality, and diabetes risk were assessed using validated questionnaires, and anthropometric and body composition measures were obtained using standardized procedures. Associations were evaluated using correlation analyses with false discovery rate correction, and multivariable linear regression was used to account for potential confounders. Night eating behavior was not associated with diabetes risk, as neither the overall night eating severity nor its subscales showed meaningful relationships with diabetes risk scores (all *p* > 0.05). Associations between night eating behavior and body composition measures were weak and did not remain significant after correction for multiple testing. In contrast, greater night eating severity, particularly nocturnal ingestions, was associated with poorer sleep-related outcomes, including longer sleep latency and increased sleep disturbances. Night eating severity remained independently associated with sleep disturbances after adjustment for age and body mass index (β = 0.336, *p* < 0.001). These findings suggest that, in young Saudi Arabian females, night eating behavior is not associated with current diabetes risk but is more closely linked to sleep disruption.

## Introduction

Type 2 diabetes (T2D) is a global health concern characterized by rising prevalence and a substantial public health burden. According to recent data, T2D is estimated to affect 578 million individuals by 2030 and increase to 700 million by 2045 worldwide^1^. The rapid rise in T2D reflects the combined influence of genetic susceptibility and modifiable lifestyle factors, including dietary behaviors, sleep patterns, and body composition. While excess adiposity and poor diet quality are well-established contributors, emerging evidence highlights the importance of when food is consumed, in addition to what and how much is eaten^2,3^.

Within this context, chrononutrition has gained increasing attention as a framework linking meal timing to circadian regulation and metabolic health. A meta-analysis highlighted that eating time is a relevant determinant of obesity and metabolic impairments^3^. Late or irregular eating patterns have been shown to disrupt circadian rhythms, impair insulin sensitivity, and alter hormonal regulators of glucose metabolism, including melatonin and cortisol. Experimental and observational studies further suggest that consuming meals later in the day, particularly close to bedtime, is associated with poorer postprandial glycemic control compared with morning intake, even when calorie and macronutrient content are standardized^4,5^. Individual chronotype may further modify these relationships by influencing both eating timing and sleep-wake patterns, yet it remains rarely assessed in studies of night eating, particularly among young adults^6^.

Within this framework, night eating, characterized by the consumption of a substantial proportion of daily energy intake during evening or nocturnal hours, has been increasingly recognized as a potential contributor to metabolic dysregulation^7^. Although the number of studies and randomized clinical trials remains limited, existing evidence suggests that late-night eating is associated with impaired glycemic control when meals are consumed close to bedtime^8^. Consistent with this, recent meta-analyses comparing morning versus evening meal timing in healthy participants consuming standardized carbohydrate meals demonstrated a higher postprandial glycemic response in the evening compared with the morning^9^.

The prevalence of night eating in Saudi Arabia and the broader Middle East is relatively high, influenced by cultural norms such as late evening meals during family or social gatherings and delayed food consumption during Ramadan. These culturally reinforced eating patterns, together with rapid urbanization and shifting sleep-wake schedules, may predispose individuals to delayed food intake. When considered alongside the high and rising prevalence of type 2 diabetes in the region, these behaviors underscore the importance of context-specific investigations that account for cultural and environmental determinants of eating behavior^10,11^. Females may experience distinct circadian and metabolic responses to mistimed eating due to hormonal fluctuations across the menstrual cycle, pregnancy, and menopause, which can influence appetite regulation, sleep patterns, and metabolic responses^12,13^. These biological factors may modify the relationship between eating timing, sleep, and metabolic health, highlighting the relevance of examining night eating behavior specifically among female populations, while avoiding assumptions of uniform vulnerability.

Sleep quality represents another critical, yet often overlooked, determinant of metabolic health. A recent meta-analysis of cohort studies demonstrated that poor sleep quality and short sleep duration are associated with an increased risk of metabolic syndrome^14^. Sleep disruption may exacerbate the metabolic consequences of unhealthy dietary patterns, contributing to a cycle of appetite dysregulation, weight gain, and insulin resistance^15,16^. Although evidence on the bidirectional relationship between night eating and sleep quality remains limited, emerging studies suggest that eating close to bedtime may negatively affect sleep duration and quality^17,18^. In parallel, short sleep duration has been consistently linked to increased risks of obesity and type 2 diabetes^19^.

Body composition, particularly excess adiposity, is a well-established risk factor for type 2 diabetes^20^. Emerging epidemiological evidence suggests that late-night eating may contribute to long-term weight gain and unfavorable body composition changes^21^. However, these associations appear less consistent in younger populations, indicating that the metabolic consequences of night eating may not be immediately detectable in early adulthood.

Accordingly, this study aimed to examine the associations between night eating behaviors, sleep quality, body composition, and type 2 diabetes risk among Saudi Arabian females. By focusing on a young, generally healthy population, this study seeks to clarify whether night eating behaviors are associated with early metabolic risk markers or are more strongly related to sleep-related outcomes.

## Method

### Study design and participants

This cross-sectional study was conducted among healthy female university students who voluntarily participated. Data collection took place at King Faisal University, and participants were recruited through social media announcements and campus posters. Eligible participants were adult females (≥ 18 years) with no prior diagnosis of diabetes, no major psychiatric or neurological disorders, and no current pregnancy or breastfeeding. Participants using medications known to substantially affect sleep, appetite, or metabolic function were excluded. Ethical approval was obtained from the King Faisal University Ethics Committee (ETHICS2732), and informed consent was obtained from all participants. The study was conducted in accordance with the principles of the Declaration of Helsinki and its subsequent amendments.

### Assessment tools

Three validated Arabic questionnaires and direct anthropometric measurements were used to examine the associations between night eating behavior, sleep quality, body composition, and type 2 diabetes risk.

### Night eating behavior

To assess night eating behavior a validated questionnaire was originally developed by Allsion and colleagues 2008^22^ and the same questionnaire was translated and validated into Arabic language^23^. This questionnaire evaluates aspects such as evening hyperphagia, nocturnal eating episodes, and awareness during nighttime eating. The night eating questionnaire (NEQ) consists of 14 items scored on a Likert scale, with higher total scores indicating more pronounced night eating behaviors. A cut-off score of ≥ 25 is commonly used to identify individuals at risk of Night Eating Syndrome (NES).

### Sleep quality

To assess sleep quality Pittsburgh Sleep Quality Index (PSQI) questionnaire was used to assess sleep quality over the past month. The PSQI was developed in 1989 by Daniel J. Buysse and colleagues^24^ and translated and validated into Arabic language^25^. This tool evaluates sleep quality over the past month through seven components: subjective sleep quality, sleep latency, sleep duration, habitual sleep efficiency, sleep disturbances, use of sleeping medication, and daytime dysfunction. Each component is scored from 0 (no difficulty) to 3 (severe difficulty), yielding a global score ranging from 0 to 21, with higher scores indicating poorer sleep quality. A global score greater than 5 is commonly used as the threshold for identifying poor sleepers.

### Type 2 diabetes risk

To estimate the risk of developing T2D within ten years a Finnish Diabetes Risk Score (FINDRISC) was used as a validated, non-invasive tool, widely used cross the world for identifying individuals at risk of type 2 diabetes^26^. The Arabic versions of the tool have been previously applied and validated in Arabic-speaking populations^27^. FINDRISC consists of eight self-reported items covering age, body mass index, waist circumference, physical activity, daily intake of fruits and vegetables, use of antihypertensive medication, history of elevated blood glucose, and family history of diabetes. Total scores categorize individuals into low (< 7), slightly elevated (7–11), moderate (12–14), high (15–20), or very high (> 20) diabetes risk. In this study, FINDRISC is referred to as the Diabetes Risk Assessment Score (DRAS).

### Anthropometric and body composition measurements

Height was measured using a wall-mounted stadiometer with participants standing barefoot. Body composition was assessed using a bioelectrical impedance analysis device (Omron Body Composition Monitor HBF-511, Omron Healthcare Co., Ltd., Kyoto, Japan). The device estimates body weight, body fat percentage, and visceral fat level using impedance measurements combined with participant age, sex, height, and weight. Waist circumference was measured using a non-elastic tape at the midpoint between the lowest rib and the iliac crest while participants were standing upright.

### Sample size calculation

Sample size was estimated a priori using Fisher’s z transformation for correlation analysis, assuming a two-sided α = 0.05, statistical power of 80%, and an anticipated moderate effect size (ρ = 0.25). The minimum required sample size was 124 participants. A total of 150 participants were recruited, exceeding this requirement and ensuring adequate statistical power.

### Statistical analyses

Statistical analyses were performed using IBM SPSS Statistics (Version 31.0; IBM Corp., Armonk, NY, USA). R software (Version 4.5.2; R Foundation for Statistical Computing, Vienna, Austria) was used for figure generation.

Descriptive statistics were presented as mean ± standard deviation or median (interquartile range) for continuous variables, and frequencies and percentages for categorical variables. Normality was assessed using the Shapiro–Wilk test and visual inspection of distributions. As several variables were non-normally distributed and questionnaire data were ordinal in nature, Spearman’s rank correlation coefficients (ρ) were used to assess associations.

The primary analysis examined the association between night eating behavior (NEQ total score) and DRAS. Secondary and exploratory analyses evaluated correlations between NEQ total and subscale scores and (i) sleep quality outcomes (PSQI global and component scores), and (ii) anthropometric and body composition measures (body weight, Body Mass Index (BMI), body fat percentage, waist circumference, and visceral fat level).

To account for multiple testing in exploratory correlation analyses, false discovery rate (FDR) correction was applied using the Benjamini–Hochberg procedure with q = 0.05. FDR correction was applied separately for the NEQ × PSQI component analyses and the NEQ × anthropometric/body composition analyses. Both unadjusted and FDR-adjusted p-values were considered when interpreting exploratory findings.

To assess whether the association between night eating behavior and sleep disturbance was independent of potential confounding, a multivariable linear regression model was fitted with PSQI Component 5 (sleep disturbances) as the dependent variable and NEQ total score as the main predictor, adjusting for age and BMI. Model assumptions were evaluated using residual diagnostics, and multicollinearity was assessed using variance inflation factors (VIF). All tests were two-tailed, with statistical significance set at *p* < 0.05 for the primary analysis.

## Results

A total of 150 Saudi Arabian female university students participated in the study. As shown in Table 1, participants were young and generally of normal weight, with low levels of central adiposity. Regarding night eating behaviors, the mean NEQ total score was 19.29 ± 5.33, suggesting low-moderate night eating tendencies, with the Nocturnal Ingestions subscale showing the highest mean (6.76 ± 2.49). The mean DRAS was 5.09 ± 3.40, indicating a generally low-to-moderate risk category for diabetes among participants. Sleep quality, assessed using PSQI components, indicated relatively elevated scores for sleep disturbances (1.60 ± 0.60) and sleep latency (1.77 ± 0.88).

**Table 1:** Baseline Characteristics of Study Participants (*N* = 150).

Variable	Mean ± SD or Median (IQR)
Demographics	
Age (years)	21 (20–23)
Anthropometrics	
Weight (kg)	54.16 ± 11.14
BMI (kg/m²)	21.10 (19–24)
Body fat (%)	32.95 ± 8.79
Waist circumference (cm)	71.96 ± 8.88
Visceral fat (level)	3.57 ± 1.35
Muscle mass (kg)	24.80 ± 2.41

*Continuous variables are presented as mean ± standard deviation (SD) if approximately normally distributed*,* or as median (interquartile range*,* IQR) if skewed.*

There was no evidence of an association between overall night eating severity and diabetes risk. The Night Eating total score was not correlated with DRAS score (Spearman’s ρ = −0.039, *p* = 0.639). Similarly, none of the NEQ subscales (morning anorexia, evening hyperphagia, nocturnal ingestions, sleep/mood disturbance, NEQ control) showed significant correlations with DRAS score (all *p* > 0.05).

**Fig. 1:**
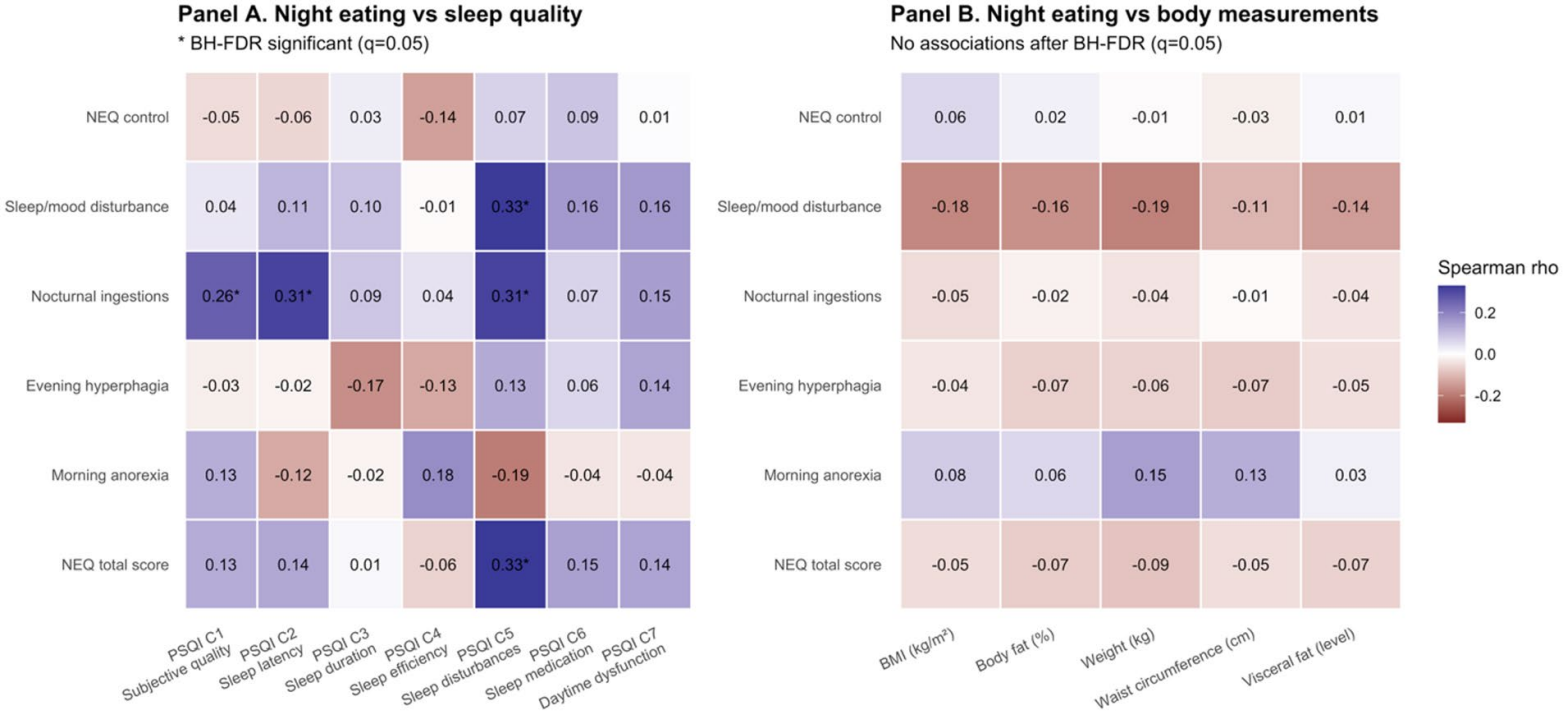
Spearman correlations between night eating (NEQ total and subscales) and (**A**) PSQI components and (**B**) body measurements. Values are Spearman’s ρ; * indicates BH-FDR significance (q = 0.05).

Spearman correlation analyses indicated that higher night eating severity was related to poorer sleep outcomes, particularly sleep disturbances. After Benjamini–Hochberg false discovery rate (BH–FDR, q = 0.05) correction across the NEQ-by-PSQI component comparisons (Fig. 1A), several associations remained significant. Higher Night Eating total score was positively correlated with PSQI Component 5 (sleep disturbances) (ρ = 0.330, *p* < 0.001). Nocturnal ingestions were positively correlated with PSQI Component 1 (subjective sleep quality) (ρ = 0.261, *p* = 0.001), PSQI Component 2 (sleep latency) (ρ = 0.311, *p* < 0.001), and PSQI Component 5 (sleep disturbances) (ρ = 0.312, *p* < 0.001). In addition, the NEQ sleep/mood disturbance subscale was positively correlated with PSQI Component 5 (sleep disturbances) (ρ = 0.328, *p* < 0.001). Other nominal associations were observed before correction, but they did not remain significant after BH–FDR adjustment.

Associations between night eating measures and body measurements (BMI, body fat percentage, weight, waist circumference, and visceral fat level) were weak overall (Fig. 1B). Although a small number of correlations reached nominal significance prior to multiple-testing correction, none remained significant after BH–FDR correction (q = 0.05) across the NEQ-by-body measurement comparisons.

A linear regression model was fitted to examine whether the association between night eating and sleep disturbances was independent of basic covariates. PSQI Component 5 (sleep disturbances) was entered as the dependent variable, with Night Eating total score, age, and BMI as predictors. The model was statistically significant (F-statistic, F(3,146) = 7.212, *p* < 0.001) and explained approximately 13% of the variance in sleep disturbances (coefficient of determination, R² ≈ 0.13). Night Eating total score remained an independent predictor of higher sleep disturbances (unstandardized regression coefficient, B = 0.037; standard error,SE = 0.009; standardized coefficient, β = 0.336; t-statistic, t = 4.348, *p* < 0.001; 95% confidence interval, CI 0.020 to 0.054), whereas age (*p* = 0.242) and BMI (*p* = 0.180) were not significant predictors. Collinearity was not a concern (variance inflation factor, VIF values ≈ 1.0).

**Table 2:** Spearman correlations between sleep quality components, diabetes risk score, and body anthropometrics.

Variable	Subjective sleep quality	Sleep latency	Sleep duration	Habitual sleep efficiency	Sleep disturbances	Use of sleep medications	Daytime dysfunction
DRAS Score	− 0.24	0.039	0.029	− 0.008	0.047	0.024	0.143
Weight (kg)	0.097	− 0.004	0.071	0.096	− 0.117	0.032	− 0.027
BMI (kg/m²)	0.032	− 0.058	0.070	0.029	− 0.020	0.011	− 0.016
Body fat (%)	0.048	− 0.009	0.077	0.034	− 0.049	0.060	− 0.039
Waist circumference (cm)	0.010	0.035	0.019	-0.014	− 0.114	0.010	− 0.081
Visceral fat (level)	0.057	0.013	0.076	-0.014	0.009	0.063	− 0.007

Values are Spearman’s rank correlation coefficients (ρ). No associations were statistically significant (*p* > 0.05); therefore, no multiple-testing correction was applied.

As presented in Table 2, DRAS scores were not significantly correlated with sleep quality or any of its PSQI components. Similarly, no significant associations were observed between sleep quality components and anthropometric measures in this cohort (all *p* > 0.05). Correlations between DRAS and anthropometric measures were examined separately and are not shown in Table 2, as these variables are components of the diabetes risk score. As expected, DRAS scores were significantly positively correlated with BMI (ρ = 0.364, *p* < 0.001), body fat percentage (ρ = 0.411, *p* < 0.001), visceral fat level (ρ = 0.400, *p* < 0.001), waist circumference (ρ = 0.375, *p* < 0.001), and body weight (ρ = 0.366, *p* < 0.001), confirming the internal consistency of the diabetes risk score.

## Discussion

This cross-sectional study examined the associations between night eating behavior, diabetes risk, body composition, and sleep quality among 150 Saudi Arabian female university students. Although the mean NEQ score indicated low-moderate night eating tendencies, night eating behavior was not associated with diabetes risk, as neither the NEQ total score nor its subscales showed meaningful relationships with DRAS. Similarly, associations between night eating measures and anthropometric or body composition variables were weak and inconsistent. In contrast, night eating particularly nocturnal ingestions was associated with poorer sleep-related outcomes. Taken together, these findings indicate that in young Saudi Arabian women, night eating behavior is more strongly linked to sleep disruption than to current metabolic risk or body composition.

The lack of association between night eating behavior and diabetes risk in this cohort aligns with several studies conducted in younger or metabolically healthy populations, in which night eating has not been consistently linked to markers of glycemic risk. In contrast, evidence linking NES to obesity, insulin resistance, and type 2 diabetes has been reported predominantly in older adults or in clinical and overweight populations^28^. Differences in age, duration of exposure to night eating behaviors, and baseline metabolic vulnerability may explain these discrepancies. In young adults with generally normal BMI and low visceral fat, the metabolic consequences of night eating may not yet be detectable, suggesting that such behaviors may precede, rather than immediately reflect, metabolic dysfunction.

In contrast to metabolic outcomes, night eating behavior demonstrated clearer associations with sleep quality. Higher overall night eating severity and more frequent nocturnal ingestions were associated with poorer subjective sleep quality, longer sleep latency, and greater sleep disturbances. These findings are consistent with prior experimental and observational research showing that nighttime food intake can interfere with circadian rhythms, delay melatonin onset, and impair sleep efficiency^17,29^. Studies conducted in Saudi and other Middle Eastern populations have similarly reported poorer sleep quality and greater daytime dysfunction among individuals exhibiting night eating behaviors^30,31^. Although the strength of these associations was modest, their consistency across multiple sleep-related domains supports a meaningful relationship between night eating and sleep disruption. Furthermore, given the known bidirectional relationship between sleep disruption and metabolic dysregulation^32,33^, persistent NES could represent a latent risk factor for future diabetes, particularly if accompanied by gradual weight gain.

Notably, the association between night eating severity and sleep disturbances remained evident after accounting for basic individual characteristics such as age and BMI. This suggests that the link between night eating and impaired sleep is not simply a consequence of body size or age but may reflect behavioral or circadian mechanisms related to the timing of food intake^34^. Within a chrononutrition framework, eating late in the biological night may conflict with endogenous circadian signals that promote fasting and sleep, thereby contributing to sleep fragmentation^35^. Nevertheless, the cross-sectional nature of the study limits causal interpretation, and it remains unclear whether night eating contributes to sleep disruption, disrupted sleep promotes night eating, or both are influenced by shared factors such as stress, irregular schedules, or circadian preference.

With respect to body composition, the absence of robust associations between night eating measures and adiposity contrasts with findings from studies conducted in older or metabolically compromised populations^36,37^. In the present cohort, the relatively narrow range of BMI and body fat percentage may have limited the ability to detect meaningful relationships. This phenomenon, often described as range restriction, is common in studies of young, healthy samples and may partly explain the weak associations observed. These findings suggest that, in early adulthood, night eating behavior alone may be insufficient to produce detectable changes in body composition without the presence of additional lifestyle or metabolic risk factors.

Associations between diabetes risk scores and anthropometric measures such as BMI and waist circumference should also be interpreted cautiously, as these variables are incorporated into, or closely related to, the scoring structure of diabetes risk tools. As such, these relationships are partly expected and do not necessarily reflect independent associations. This highlights the importance of considering the methodological characteristics of composite risk scores when interpreting correlational findings and supports the use of complementary metabolic biomarkers in future research.

### Strengths and limitations

A key strength of this study is its focus on an underrepresented population. To our knowledge, this study is among the first in Saudi Arabia to comprehensively examine night eating behavior in relation to sleep quality, body composition, and diabetes risk among young adult females using validated assessment tools. The use of standardized questionnaires and directly measured anthropometric data enhances data quality and reduces reporting bias. Additionally, the inclusion of multivariable regression analysis allowed partial control for confounding factors and strengthened the interpretation of associations between night eating and sleep disturbances.

Several limitations should be acknowledged. The cross-sectional design precludes causal inference and limits conclusions regarding directionality. Night eating behavior and sleep quality were assessed using self-reported instruments, which may be subject to recall or social desirability bias. The sample size, while adequate for detecting moderate associations, may have limited power to detect smaller effects, which is common in observational studies of relatively homogeneous young populations. Moreover, although some associations were statistically significant, their strength was modest and should be interpreted cautiously. Chronotype, dietary intake, and physical activity were not assessed and may represent important unmeasured confounders. Future longitudinal and interventional studies addressing these factors are warranted.

## Conclusion

In this cohort of young Saudi Arabian females, night eating behavior was not associated with diabetes risk and showed no robust associations with body composition after correction for multiple testing. In contrast, night eating particularly nocturnal ingestions was associated with poorer sleep-related outcomes, and overall night eating severity independently predicted sleep disturbances after adjustment for age and BMI. These findings indicate that night eating in this population may primarily reflect sleep disruption rather than current metabolic risk.

## Data Availability

The datasets generated and/or analyzed during the current study are available from the corresponding author on reasonable request.

## Abbreviations

NEQ: Night eating questionnaire
NES: Night eating syndrome
PSQI: Pittsburgh sleep quality index
DRAS: Diabetes risk assessment score
FINDRISC: Finnish diabetes risk score
BMI: Body mass index
SD: Standard deviation
IQR: Interquartile range
T2D: Type 2 diabetes
